# The psoriasis-associated deletion of late cornified envelope genes *LCE3B* and *LCE3C* has been maintained under balancing selection since Human Denisovan divergence

**DOI:** 10.1186/s12862-016-0842-6

**Published:** 2016-12-05

**Authors:** Petar Pajic, Yen-Lung Lin, Duo Xu, Omer Gokcumen

**Affiliations:** Department of Biological Sciences, University at Buffalo, Cooke 639, Buffalo, NY 14260 USA

**Keywords:** Copy number variation, Genomic structural variants, Atopic dermatitis, HLA, Defensins, Neanderthal, LCE3A, Human evolution

## Abstract

**Background:**

A common, 32kb deletion of *LCE3B* and *LCE3C* genes is strongly associated with psoriasis. We recently found that this deletion is ancient, predating Human-Denisovan divergence. However, it was not clear why negative selection has not removed this deletion from the population.

**Results:**

Here, we show that the haplotype block that harbors the deletion (i) retains high allele frequency among extant and ancient human populations; (ii) harbors unusually high nucleotide variation (π, *P* < 4.1 × 10^−3^); (iii) contains an excess of intermediate frequency variants (Tajima’s D, *P* < 3.9 × 10^−3^); and (iv) has an unusually long time to coalescence to the most recent common ancestor (TSel, 0.1 quantile).

**Conclusions:**

Our results are most parsimonious with the scenario where the *LCE3BC* deletion has evolved under balancing selection in humans. More broadly, this is consistent with the hypothesis that a balance between autoimmunity and natural vaccination through increased exposure to pathogens maintains this deletion in humans.

**Electronic supplementary material:**

The online version of this article (doi:10.1186/s12862-016-0842-6) contains supplementary material, which is available to authorized users.

## Background

Genomic structural variants (SVs), which are deletions, duplications, inversions and translocations of genomic segments, account for the majority of variable base pairs in primate genomes [1, 2]. Because of their sheer size, SVs can have strong effects on gene function and regulation if they overlap with protein-coding (eg, [3]) or regulatory sequences (eg, [4]). Indeed, several studies have revealed important roles that SVs play in human evolution [2, 5] and adaptation [6, 7].

Disruption of a gene’s function by deletion of its coding sequence likely reduces fitness and predisposes humans to several genetic disorders (eg, [8–10]). Consistent with this notion, deletion variants among humans are distributed significantly away from coding sequences [11] and most exonic deletions are found in very low frequencies in human populations [12]. In a recent study, we searched for unusually old deletion variants that affect coding sequences. Specifically, we identified exonic deletions that evolved before Human-Neanderthal divergence (>500-1,000KYA) [13]. We surmised that it is unlikely for a loss-of-function gene deletion to be maintained for this long, especially under negative selection. Thus, we hypothesized that a number of these ancient deletion variants have been evolving under balancing selection.

Balancing selection has enjoyed a renewed interest in the evolutionary genomics community. In its most basic form, balancing selection can be thought of as the combination of adaptive forces that maintain variation longer than expected under neutrality [14, 15]. Based on the analyses of recently available human and nonhuman primate genomes, several variants have been shown to be evolving under long-term balancing selection in the human-chimpanzee lineage [16, 17]. In addition, multiple instances of balancing selection within the human gene pool have been reported in the last decade [18–23].

In this paper, we investigate the evolution of an ancient gene deletion (*LCE3BC* deletion). This ~32kb deletion variant overlaps 2 genes, *LCE3B* and *LCE3C*, which are both involved in skin tissue repair. We recently showed that this deletion variant is derived in the *Homo* lineage and that the deletion is present in the Denisovan genome, but absent in the Neanderthal genome [13]. In the same study, we were able to rule out archaic introgression and concluded that incomplete lineage sorting best explains the observed allele sharing at this locus. The deletion is very common in humans, reaching up to 70% in some European populations. Moreover, this deletion has been strongly associated with psoriasis susceptibility, with odds ratios ranging from 1.3 (The Italian population) to 1.9 (The Chinese population) [24–27]. However, the adaptive reasons for why this deletion remains in the population are unknown.

## Results

**Fig. 1:**
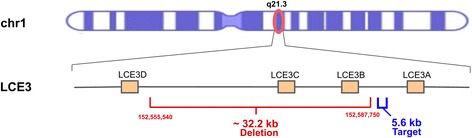
Genomic location of the *LCE3BC* deletion. *Top*: Location of the *LCE3BC* deletion on chromosome 1q21.3 (*red circle*). *Below*: The zoomed-in look at the region harboring the *LCE3BC* deletion. The red bar shows where the deletion occurs (Hg19, chr1:152,555,540–152,587,750). The blue bar represents the 5.6 kb “target” (chr1: 152,587,904–152,593,549) sequence that we used to conduct the majority of the population genetics analyses in this study

The *LCE3BC* deletion spans slightly more than 32kb in the human reference genome and overlaps with two conserved, protein coding genes, *LCE3B* and *LCE3C* (Fig. 1). We manually confirmed that this deletion has been shared with Denisovans, but not with Neanderthals (Additional file 1: Figure S1). We then determined single nucleotide variants that are in high linkage disequilibrium (R^2^ > 0.9) with the *LCE3BC* deletion among 2504 human genomes independent of population ancestry [28] (Additional file 2: Table S1). Using this dataset, we identified a haplotype block that comprises the *LCE3BC* deletion and its flanking sequences extending to 6.6kb downstream and 5.5kb upstream (Additional file 1: Figure S2).

**Fig. 2:**
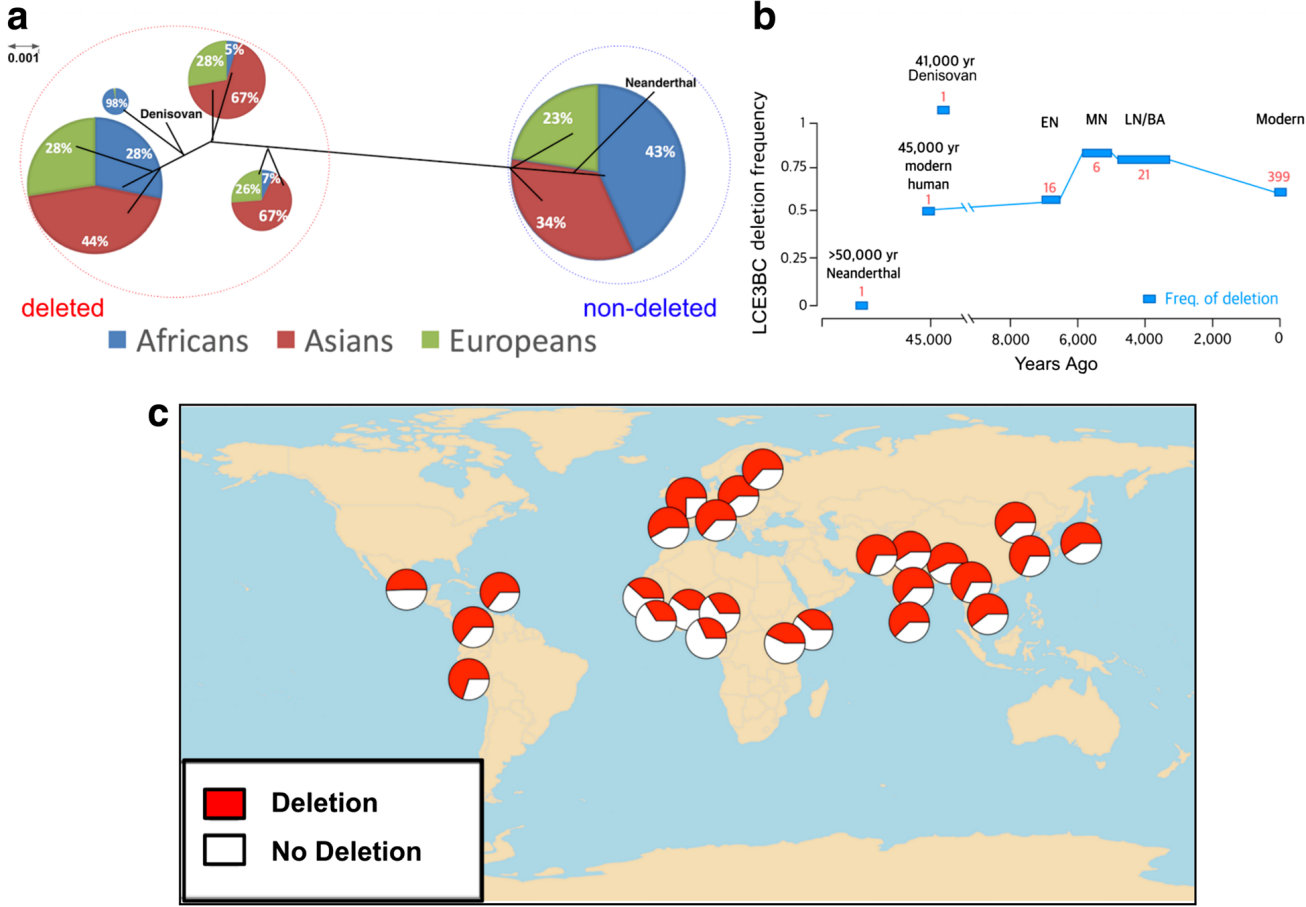
Global haplotype variation of the *LCE3BC* locus and allele frequency of the *LCE3BC* deletion in modern and ancient populations. **a** Simplified phylogenetic tree based on 5008 human haplotypes from the 1000 Genomes Project. We constructed the tree using the single nucleotide variants downstream of the deletion (also see Additional file 1: Figure S3). Haplotypes separated into two distinct haplogroups, which segregate into haplotypes that carry the deletion (*deleted*, red circle) and those that do not carry the deletion (*non-deleted*, blue circle). Frequency pie charts were superimposed to the tree for an overview of the distribution of the major haplotype groups across continental populations (Africans: [ACB, ASW, ESN, GWD, LWK, MSL, YRI], Asians: [BEB, CDX, CHB, CHS, GIH, ITU, JPT, KHV, PJL, STU] and Europeans: [CEU, FIN, GBR, IBS, TSI]). **b** Frequency distribution of the *LCE3BC* deletion in ancient human genomes. The x-axis indicates the date of the samples. The y-axis indicates the allele frequency of the deletion (as imputed by the frequency of tag single nucleotide variants rs6693105). The population acronyms are EN: Early Neolithic European; MN: Middle Neolithic European; BA: Bronze Age European. Each blue box indicates the ancient population and the length of the box spans the estimated age of the samples. The sample size of each population is indicated by the red number on the top or bottom of each box. **c** The geographic distribution of the *LCE3BC deletion* allele frequency. The red color indicates the frequency of the haplotypes in each population carrying the deletion, whereas white are haplotypes without the deletion

We constructed a maximum likelihood tree of the variation in the region downstream of the *LCE3BC* deletion (target region) among 5008 modern human haplotypes, as well as Denisovan, Altai Neanderthal, Chimpanzee and Rhesus Macaque haplotypes (Fig. 2a, Additional file 1: Figure S3). We found a clear separation of two haplogroups (named hereon *deleted* and *non-deleted*) with perfect segregation of haplotypes carrying the deletion and those that do not. Furthermore, the Denisovan haplotype, which carries the deletion, clusters with the *deleted* human haplotypes, while the Neanderthal haplotype, which does not carry the deletion, clusters with the *non-deleted* haplotypes (Fig. 2a). This observation provides further support that the *LCE3BC* deletion evolved before Human-Denisovan split and remains variable since then.

We then traced back the allele frequency of rs6693105, which tags the *LCE3BC* deletion in all modern humans (R^2^ = 0.95), in recently available ancient genomes (Additional file 3: Table S2). We found that the genome of the 45,000 year old individual from Central Asia (Altai) [29] is heterozygous for the deletion allele (Fig. 2b). In fact, we were able to directly confirm the loss of read-depth due to heterozygous deletion in this sample (Additional file 1: Figure S1). We also found that the deletion haplotype has always been found in high frequency in ancient European populations (Fig. 2b). The *LCE3BC* deletion is very common in extant human populations as well, reaching major allele status in all non-African populations (Fig. 2c).

To explain the evolutionary forces that shape this ancient (older than Human-Denisovan divergence) and very common allele (>50% frequency in most populations), we consider three scenarios. First, it is plausible that the *LCE3BC* deletion evolved under neutrality (eg, [30]). Indeed, in a recent study, we provided evidence that a neighboring gene, *FLG*, harbors neutrally evolving common loss-of-function variants [31]. In this scenario, we would expect the haplotype block carrying the deletion variant to show no significant deviation in tests of neutrality, when compared to neutral regions of the genome. Second, we considered positive selection, which would increase the frequency of this variant in human populations. Such cases were reported for other loss of function variants [32]. In this scenario, we expect increased population divergence (*F*_*ST*_) [33] and deviation from expected homozygosity (delta integrated homozygosity score (**∆**iHH)) [34], but reduced nucleotide diversity (*π*). Third, we considered balancing selection, where we expect high π and a deviation from the expected frequency site spectrum (eg, positive values of Tajima’s D).

**Fig. 3:**
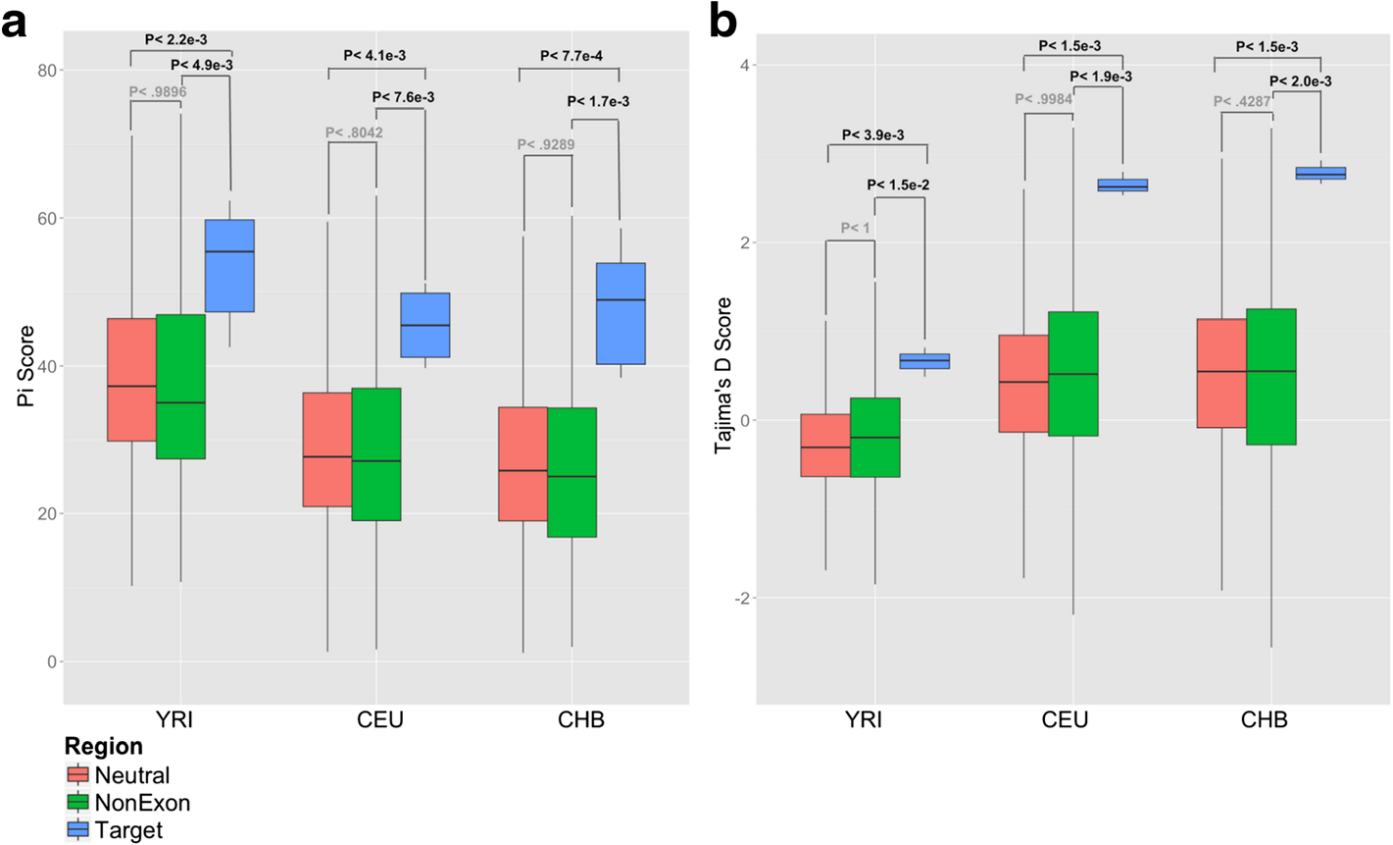
Population genetics summary statistics (**a**) Y-axis of the boxplot shows the nucleotide diversity (π) and (**b**) Tajima’s D in three different populations (CEU: Central Europeans from Utah; CHB: Han Chinese from Beijing; and YRI: Yoruba from Ibadan Nigeria). The red boxes represent all regions of the genome that are predicted to be evolving under neutrality, whereas the blue boxes represent the data points from the *LCE3BC* haplotype block. The green boxes represent π and Tajima’s D scores calculated for the size-matched downstream regions of *non-exonic* ancient deletions. These deletions are comparable to the *LCE3BC* deletion in the sense that they were also found to have evolved before Human Neanderthal/Denisovan divergence and that they are similarly high in allele frequency [58]. *P*-values (Wilcoxon Test) are shown on top of the comparisons

To test the above hypotheses, we compared different population genetics statistics for the *LCE3BC* haplotype block to those calculated for neutrally evolving regions as defined by Arbiza et al. [35] (Additional file 4: Table S3). We found no significant increase of **∆**iHH or F_ST_ in the *LCE3BC* haplotype block as compared to neutral regions (Additional file 1: Figure S4). In contrast, we observed that nucleotide diversity (π) in the *LCE3BC* haplotype block is at least 2 fold higher than neutral regions (Wilcoxon Test, CEU - *P* < 4.1 × 10^−3^; CHB - *P* < 7.7 × 10^−4^; YRI - *P* < 2.2 × 10^−3^) (Table 1, Fig. 3a) Similarly, Tajima’s D statistics in the *LCE3BC* haplotype block was significantly higher than neutral regions in *all* populations tested (Wilcoxon Test, CEU - *P* < 1.5 × 10^−3^; CHB - *P* < 1.5 × 10^−3^; YRI - *P* < 3.9 × 10^−3^) (Table 1, Fig. 3b). Tajima’s D compares the pairwise differences between haplotypes and the number of segregating sites [36]. In practice, positive values of Tajima’s D indicate an excess of intermediate frequency variants, which is a hallmark of balancing selection. Last but not least, we found that the region 10kb downstream of the *LCE3BC* deletion is within the 10^th^ percentile of genome-wide distribution of pairwise time to most recent common ancestor as measured by Tsel method [37].

**Table 1:** Summarized mean and standard deviation values for Tajima’s D and Pi for different population and for the target and neutral regions, as well as regions adjacent to comparable nonexonic ancient deletions

Population	Region	Tajima’s D	Pi
CEU	Target	2.65+/−0.13	45.48 +/−5.62
	Neutral	0.41+/−0.87	29.65 +/−12.56
	Non-Exon	0.49 +/−1.05	31.32 +/−35.12
CHB	Target	2.97+/−0.13	48.00 +/−8.67
	Neutral	0.49 +/−0.96	27.73 +/−12.53
	Non-Exon	0.46 +/−1.19	29.21 +/−35.38
YRI	Target	0.66+/−0.16	53.58 +/−8.14
	Neutral	−0.27+/−0.54	39.30 +/−13.21
	Non-Exon	−0.17 +/−0.70	40.29 +/−31.30

These standard tests of neutrality can be affected by the mutation rate, as well demographic history [38]. One specific worry was whether preselecting a known “ancient” deletion that is shared with an archaic hominin species may bias our results. For example, it is possible that increased π may be due to the fact that the variation in this locus is older than many other parts of the genome. To address this, we compared Tajima’s D and π observed for the *LCE3BC* haplotype block with those calculated for size matched regions downstream of 340 other “ancient”, non-exonic deletions. These deletions were previously found to be shared with Neanderthals and/or Denisovans due to incomplete lineage sorting and also present in high allele frequency in contemporary human populations. As such, they are comparable to the *LCE3BC* deletion both for their age and allele frequency. This analysis confirmed our previous observations that π (Wilcoxon Test, CEU - *P* < 7.6 × 10^−3^; CHB - *P* < 1.7 × 10^−3^; YRI - *P* < 4.9 × 10^−3^) and Tajima’s D (Wilcoxon Test, CEU - *P* < 1.9 × 10^−3^; CHB - *P* < 2.0 × 10^−3^; YRI - *P* < 1.5 × 10^−2^) calculated for the LCE3BC haplotype block is significantly higher than those calculated for other ancient deletion haplotype blocks (Fig. 3a and b). We also considered the potential impact of repetitive sequences to variant calling quality. As such, we confirmed all our main results, omitting variants coinciding with repetitive sequences (Additional file 1: Figures S5–S7).

## Discussion

In this study, we analyzed the haplotype block specifically because it harbors an ancient, exonic and disease-associated deletion variant. Our analyses of the haplotypic variation harboring this deletion best fits a model where the *LCE3BC* deletion has been maintained under balancing selection in human populations. What remains an open question is the adaptive pressure(s) on the *LCE3B* and *LCE3C* gene functions at the organismal and ecological levels.

The phenotypic impact of the *LCE3BC* deletion has been discussed extensively, especially within the context of psoriasis biology (reviewed in [39]). Briefly, *LCE3B* and *LCE3C* coding sequences, which remain highly conserved across mammals (Additional file 1: Figure S8), are both active in skin barrier repair. As such, their expression is mostly confined to injured skin [40] and deletion of these genes likely leads to inefficient skin barrier repair. In addition, a likely regulatory region is also eliminated by the deletion [41] (Additional file 1: Figure S8). It also appears that the deletion haplotype leads to a significant increase in *LCE3A* expression in sun-exposed skin (Additional file 1: Figure S9), which may be a partial compensatory response to loss of *LCE3B* and *LCE3C* activity.

It is unknown how the lack of LCE3B and LCE3C activity due to their deletion leads to psoriasis susceptibility. Variants that are related to skin structure (eg, *LCE3BC* deletion [24]) and variants related to immune function [42–44] are independently associated with psoriasis. Moreover, epistatic interactions between HLA-C*06 and *LCE3BC* loci were reported within the context of psoriasis [24, 25]. Briefly, it appears that the *LCE3BC* deletion leads to slower repair of the epidermal barrier, which in turn leads to increased exposure to environmental antigens and pathogens. The higher level of exposure consequently leads to higher activity of immune elements, and occasionally pathological autoimmune response.

Based on the above-described mechanism, Bergboer et al. [39] hypothesized that the *LCE3BC* deletion would be favored to increase the effectiveness of the acquired immunity system (ie, natural vaccination), with the drawback of increasing the susceptibility to autoimmune disorders. In fact, it is plausible that the effect of the *LCE3BC* deletion on the immune system may be more than skin-deep, as the deletion was also associated with more systemic autoimmune disorders, such as psoriatic arthritis [45] and lupus [46]. Our findings presented here are concordant with this hypothesis.

## Conclusion

The recent availability of high quality whole genome data at the population level provides novel opportunities to investigate complex evolutionary forces that shape disease susceptibility loci without ascertainment bias. Using such an approach, we provide multiple lines of evidence that a common, 32kb deletion strongly associated with psoriasis has evolved under balancing selection in the human lineage [47]. Our study presents empirical evidence that balancing selection on the *LCE3BC* deletion contributes to this very interesting dynamic. Our results will also contribute to the renewed discussion in the community on balancing selection maintaining advantageous diversity in human populations [17, 18, 22].

## Methods

This 5.6kb region was selected based on the linkage disequilibrium between the single nucleotide variants and the deletion (R^2^ > 0.8) (Additional file 2: Table S1). Therefore, we surmised that this region is the haplotype block that harbors the deletion and we expect that the characteristics of the genetic variation within this region would inform us with regards to the evolutionary forces that shape the deletion as well. To investigate genetic variation within the *LCE3BC* haplotype block, we utilized the 1000 Genomes Project dataset, which includes 2504 human genomes across 26 populations [28], multiple ancient genomes [29, 48–50], chimpanzee and rhesus macaque reference haplotypes [51, 52] (Additional file 2: Table S1 and Additional file 3: Table S2). To do this, we used a custom pipeline, which is available at *github* (https://github.com/duoduoo/VCFtoTree).

It has been shown that single nucleotide variation calling in next generation resequencing data may be affected in regions with duplicated or repeat-rich regions of the genome. We previously verified that all the single nucleotide variants that we used in our analyses have passed the 1000 Genomes quality filters (Quality score = 100) [11]. However, to further ensure that our analyses are not biased with repeated segments of the genome, we checked the target region where we conducted our analyses for presence of segmental duplications and other repetitive sequences. In addition, we used BLAT to check sequence uniqueness of this region. These analyses confirmed that there are no segmental duplications within our target region, but detected an L1 element (Fig. 1). Based on this search, we found an L1 element within the target region (Additional file 1: Figure S5, chr1:152,587,904–152,590,051). Since, it is plausible that this element can cause spurious single nucleotide variant calls, creating increased heterozygosity, we re-conducted our main analyses on the newly constructed target region (chr1:152,590,052–152,593,549), omitting the LINE element. The results confirm that our original conclusions remain unchanged, based on identical differentiation of haplogroups on the maximum likelihood tree (Additional file 1: Figure S6) and unchanged results for Tajima’s D and *π* (Additional file 1: Figure S7).

We constructed a maximum likelihood tree using RAxML [53] with single nucleotide variants downstream of the deletion [Hg19-chr1: 152,587,904–152,593,549]. The alignments can be found as a supplementary file as well as on our website (gokcumenlab.org/data-and-codes/). We used Dendroscope [54] program for visualization. We constructed a rooted maximum likelihood tree using PhyML (HKY85 model) and we bootstrapped with 1000 replicates for branch support. We used values calculated by the 1000 Genomes Selection Browser [55] for comparing multiple population genetics parameters. To visually inspect the deletion variation in archaic humans, we used Integrated Genome Viewer [56]. We used ENCODE and Gtex databases to search for functional variants within the LCE3BC haplotype block. For compiling allele frequencies in ancient genomes, we used PLINK [57]. We used Python and R for custom bioinformatic and statistical analyses of the genetic variation. The R codes and processed datasets to replicate our core analyses are available as supplemental files (Additional File 5: File S1, Additional File 6: File S2) as well as on our website (http://gokcumenlab.org/data-and-codes/).

## Additional files

Additional file 1: Supplementary Figures. (PDF 1236 kb)
Additional file 2: Table S1. Linkage disequilibrium between LCE3 BC deletion and adjacent SNPs. (XLSX 64 kb)
Additional file 3: Table S2. rs6693105 (ie, linked with L CE3BC deletion) a llele frequency in ancient human samples. (XLSX 39 kb)
Additional file 4: Table S3. Tajima’s D, Pi, Fst, ΔiHH for Neutral, ancient and Target regions. (XLSX 7546 kb)
Additional file 5:
**File S1.** Codes. (TXT 2 kb)
Additional file 6:
**FIle S2.** Input file for generating the World Map. (TXT 1 kb)

## Abbreviations

ACB: African Caribbeans in Barbados
ASW: Americans of African Ancestry in SW USA
*BA*: Bronze Age European
BEB: Bengali from Bangladesh
*bp/kb*: Basepairs/Kilobases
CDX: Chinese Dai in Xishuangbanna, China
CEU: Central Europeans in Utah
CHB: Han Chinese in Bejing, China
*chr*: Chromosome
CHS: Southern Han Chinese
*EN*: Early Neolithic European
ESN: Esan in Nigeria
FIN: Finnish in Finland
*FLG*: Filaggrin
GBR: British in England and Scotland
GIH: Gujarati Indian from Houston, Texas
GWD: Gambian in Western Divisions in the Gambia
*HLA*: Human leukocyte antigen
IBS: Iberian Population in Spain
*IGV*: Integrative genome viewer
ITU: Indian Telugu from the UK
JPT: Japanese in Tokyo, Japan
KHV: Kinh in Ho Chi Minh City, Vietnam
*LCE3A/LCE3B/LCE3C/LCE3BC*: Late cornified envelope 3 A/B/C/BC
*LD*: Linkage disequilibrium
LWK: Luhya in Webuye, Kenya
*MN*: Middle Neolithic European
MSL: Mende in Sierra Leone
*PhyML*: Phylogenetic estimation using maximum likelihood
PJL: Punjabi from Lahore, Pakistan
*SNPs*: Single nucleotide polymorphisms
STU: Sri Lankan Tamil from the UK
*SVs*: Structural variants
TSI: Toscani in Italia
YRI: Yoruba in Ibadan, Nigeria
