# Supplementary material for: The psoriasis-associated deletion of late cornified envelope genes LCE3B and LCE3C has been maintained under balancing selection since Human Denisovan divergence
Source: BMC Evol Biol. 2016 Dec 5;16:265. doi: 10.1186/s12862-016-0842-6 (PMC5139038; doi:10.1186/s12862-016-0842-6)

The psoriasis-associated deletion of late cornified envelope genes *LCE3B* and *LCE3C* has been maintained under balancing selection since Human Denisovan divergence

Petar Pajic[1]*, Yen-Lung Lin[1]*, Duo Xu[1], Omer Gokcumen[1]
Department of Biological Sciences, University at Buffalo, Buffalo, NY.

***The authors contributed equally to this work**

Corresponding Author
Omer Gokcumen
Department of Biological Sciences
University at Buffalo
Cooke 639
Buffalo, NY 14260
omergokc@buffalo.edu

KEYWORDS: **Copy number variation, genomic structural variants, atopic dermatitis, HLA, defensins, Neanderthal, LCE3A, Human Evolution**

SUPPLEMENTARY FILES

Figure S1. Integrative Genome Viewer (IGV) screenshot shows the sequence alignments in Neanderthal, Denisovan and 45k Siberian genomes for the LCE3BC locus. The bottom blue boxes represent the genes in the LCE3 region. The green line shows the breakpoints of the variable *LCE3BC deletion* among humans. The small grey lines are individual reads from Altai Neanderthal (Top), Denisovan (Middle), and the 45,000 year old Siberian Human (Bottom) genomes mapping to the human reference genome (Hg19). The histogram above each sample indicates the read-depth at that location.

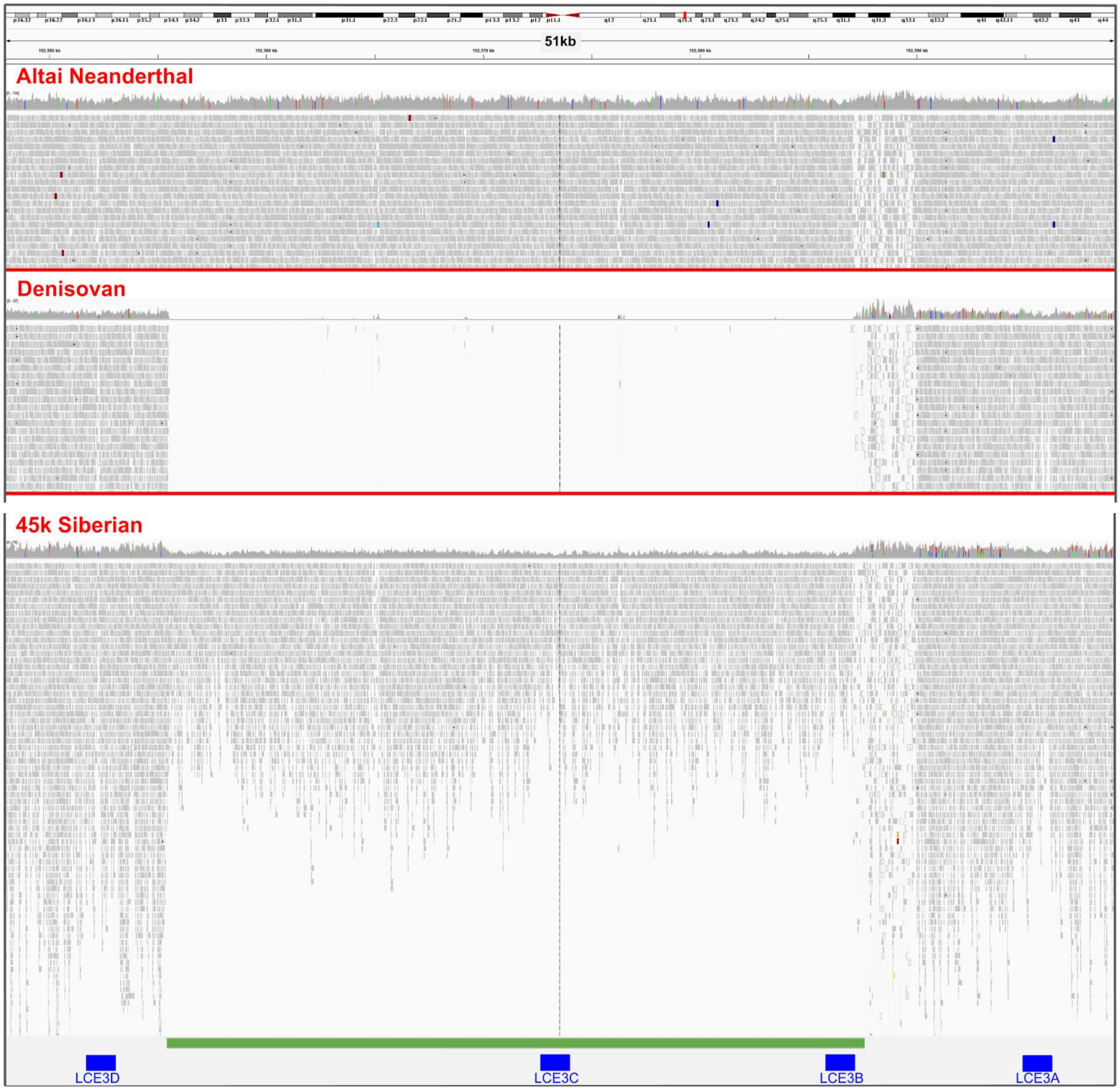

Figure S2. Linkage Disequilibrium (LD) scores within and around the *LCE3BC* deletion. The X-axis represents the chromosomal location, while the y-axis is the R^2 score. The blue arrows represent the location of the *LCE3BC* deletion. The single nucleotide variants with high level (R^2>0.9) are shown in red dots and found to range from 5.5kb upstream to 6.6kb downstream of the *LCE3BC* deletion. The 5.6kb target region indicated by the red bracket was used for majority of the population genetic analyses. This target region also harbors the SNP that was used for interrogating ancient genomes, rs6693105.

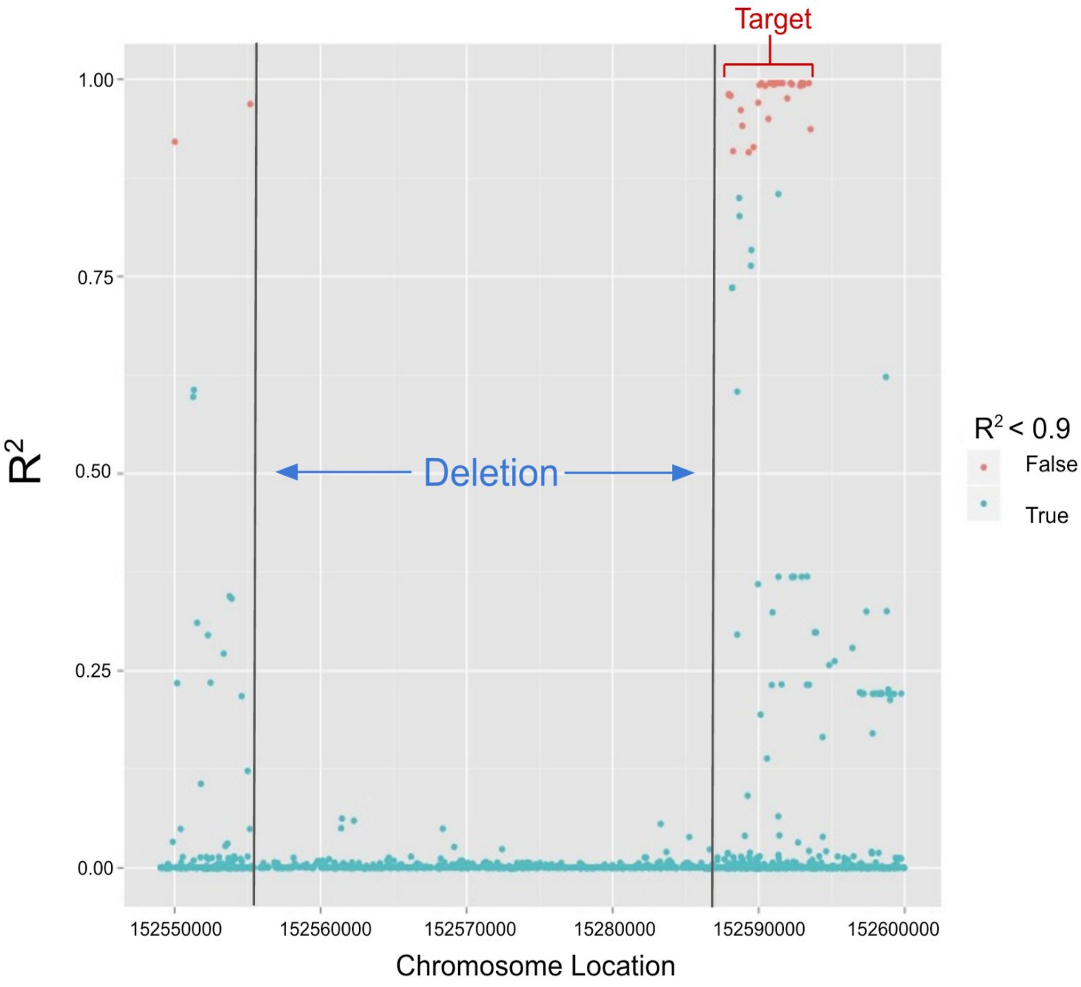

Figure S3. (a) Maximum likelihood haplotype tree constructed using 1000 Genomes Phase 3 dataset. This input file for the tree is the same as the one used to create Figure 2c, with the addition of the Chimpanzee (*Pan troglodytes*). The Chimp grouped with the “non-deleted” haplotype group, represented by the blue circle. **(b)** Rooted maximum likelihood haplotype tree constructed using a select 24 haplotypes from 1000 Genomes Phase 3 dataset, representing major branches. Rhesus Macaque (rheMac3) was used as an outgroup. The blue haplotypes, Altai Neanderthal, and Chimpanzee grouped together as *non-deleted*. The red haplotypes and Denisovan cluster with the deleted haplogroup. Parametric bootstrap branch support values were included on the primary branches.

a.

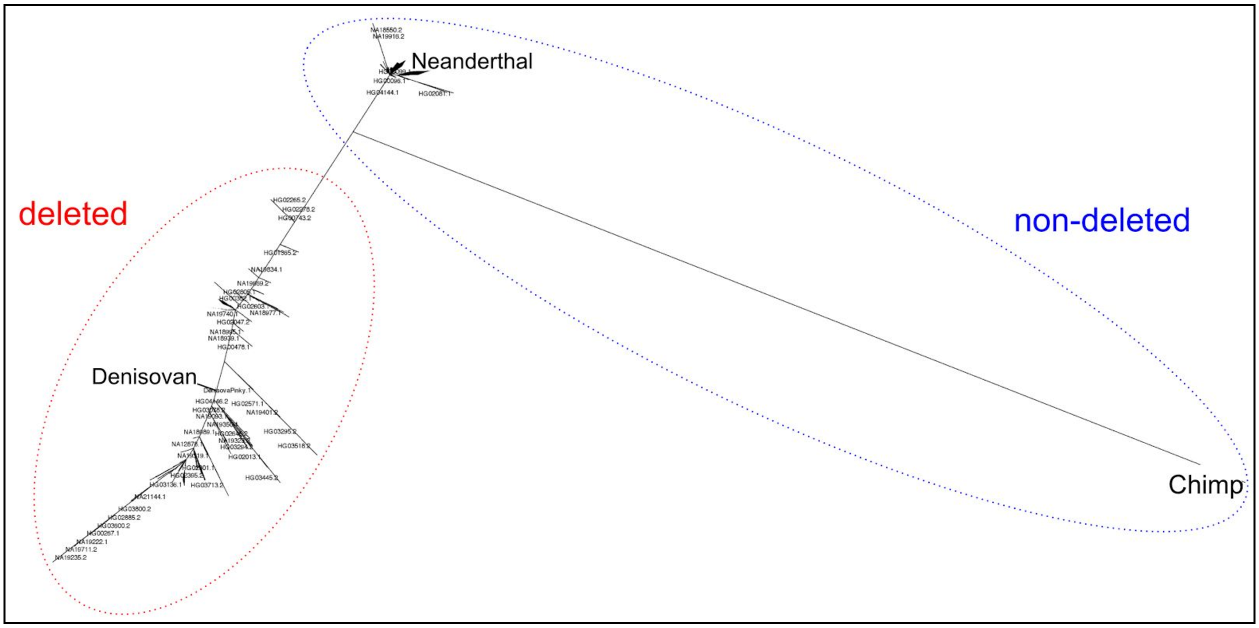

b.

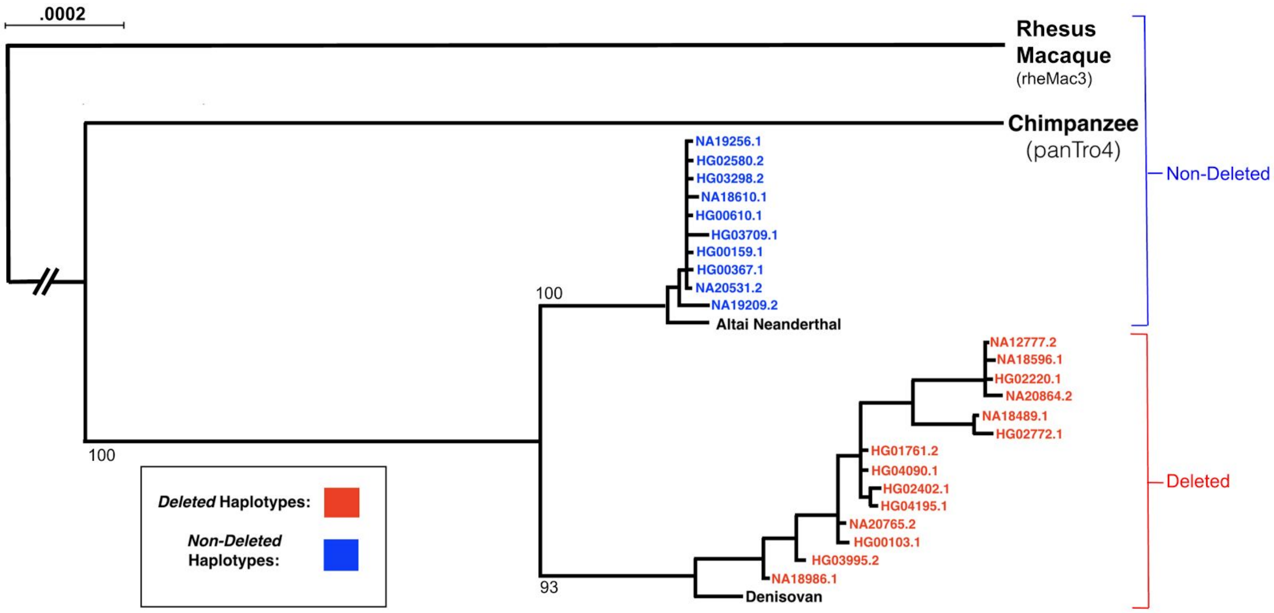

Figure S4. (a) Pairwise population differentiation (F_{ST}, y-axis) between 3 populations; CEU vs. YRI; CHB vs. CEU; and YRI vs. CHB. The populations considered here are CEU: Central Europeans from Utah; CHB: Han Chinese from Beijing; and YRI: Yoruba from Ibadan Nigeria (x-axis). The red box represents the neutral regions on chromosome 1 with the blue representing the previously consistent "target" region. This is consistent with balancing selection acting on this locus. **(b)** Integrated haplotype homozygosity (ΔiHH) (y-axis) for the populations considered here; CEU: Central Europeans from Utah; CHB: Han Chinese from Beijing; and YRI: Yoruba from Ibadan Nigeria (x-axis). Data was taken from 1000 Genomes Dataset.

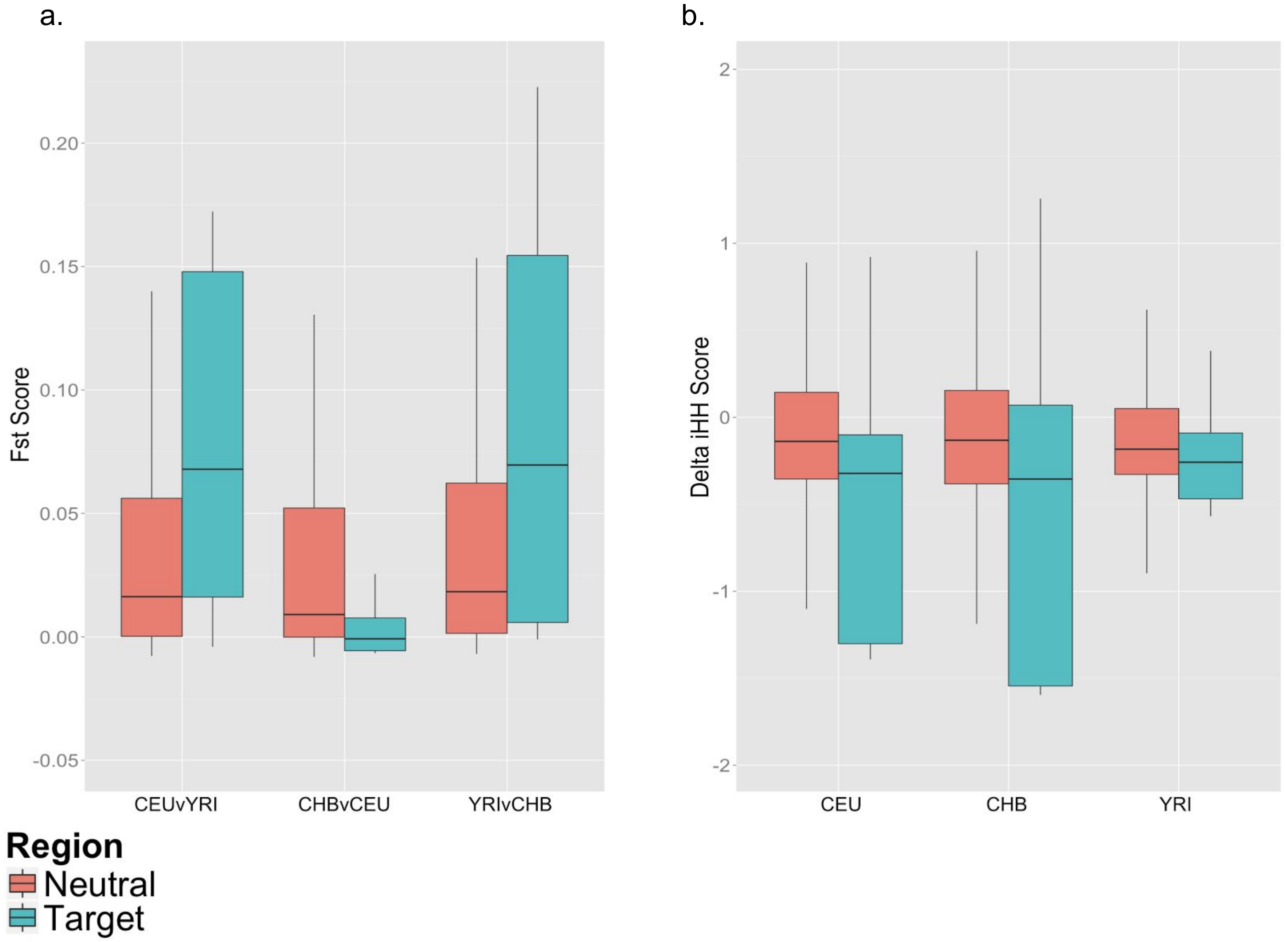

Figure S5. Location of the L1 (LINE) element (chr1: 152,587,904-152,590,051) within the "target" haplotype block (red), with respect to the LCE3BC deletion (blue). The original target region (152,587, 904 - 152,593,549; green), has been reconstructed for analysis, avoiding the LINE element (152,590,052-152,593,549; purple).

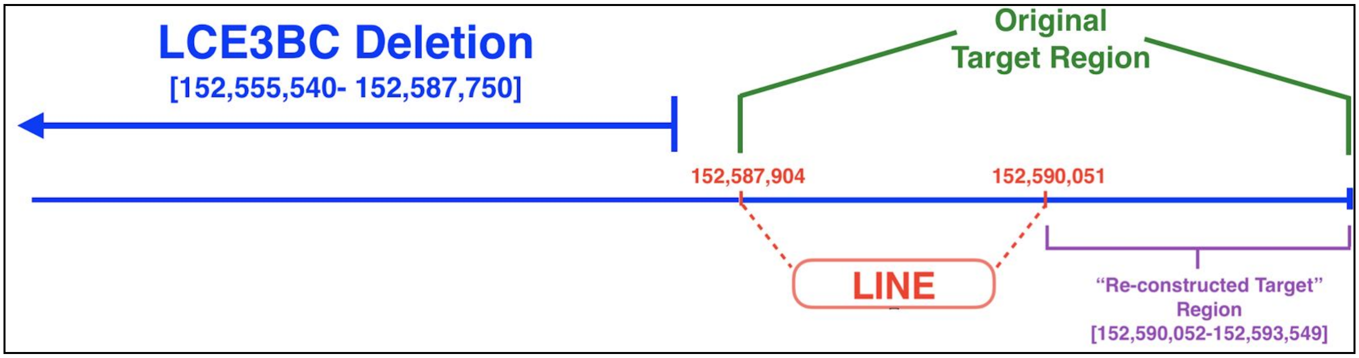

Figure S6. Re-constructed maximum likelihood haplotype tree using 1000 Genomes Phase 3 dataset to avoid the L1 element. This tree compares to the one previously constructed in **Figure 2**, with no changes other than branch swapping. The same haplotypes group with the same haplogroups as expected.

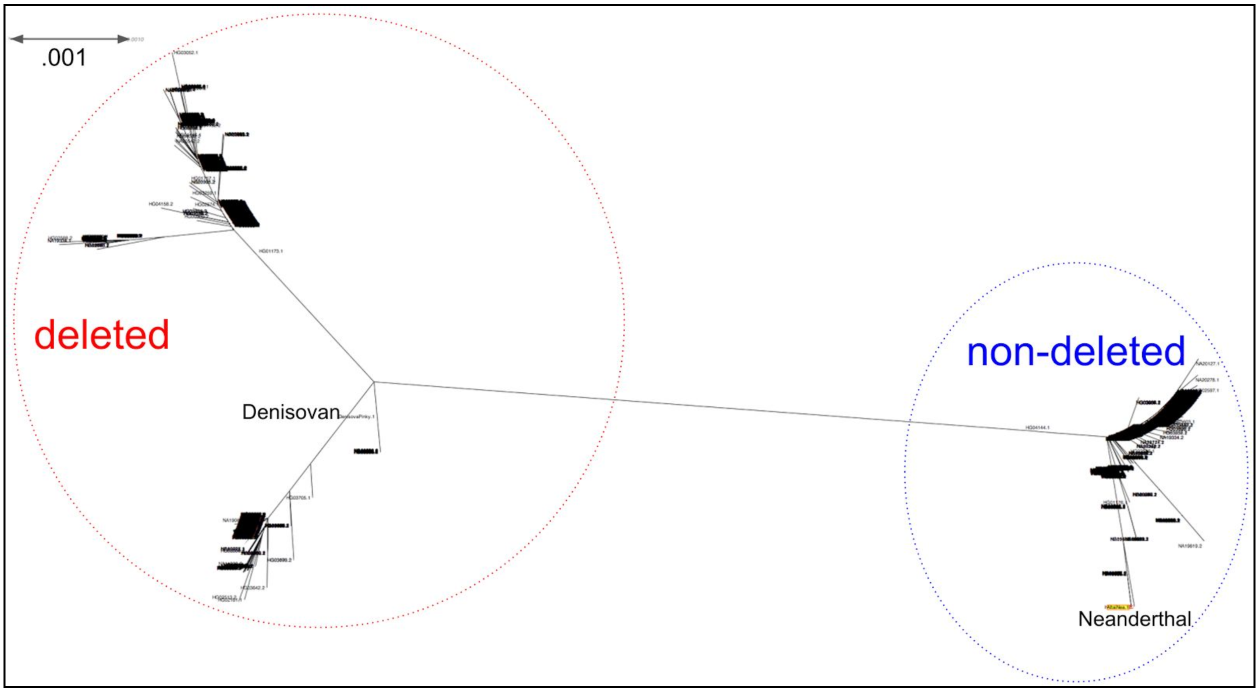

Figure S7. (a) Tajima's D score in three different populations (CEU: Central Europeans from Utah; CHB: Han Chinese from Beijing; and YRI: Yoruba from Ibadan Nigeria). The red boxes represent all regions of the genome that are predicted to be evolving under neutrality, whereas the blue boxes represent the data points from the re-constructed *LCE3BC* haplotype block omitting the region harboring the LINE element. **(b)** Pi score in three different populations (CEU: Central Europeans from Utah; CHB: Han Chinese from Beijing; and YRI: Yoruba from Ibadan Nigeria). The red boxes represent all regions of the genome that are predicted to be evolving under neutrality. The blue boxes represent the data points from the re-constructed *LCE3BC* haplotype block omitting the region harboring the LINE element. The green boxes represent π and Tajima's D scores calculated for the size-matched downstream regions of *non-exonic* ancient deletions.

TajD P-values:
(**YRI;** Target:Neutral; 1.6e-2, Target: NonExon; 3.8e-2, Neutral:NonExon; 1.032e-5)
(**CEU;** Target:Neutral; 7.5e-3, Target: NonExon; 8.9e-3, Neutral:NonExon; 1.6e-3)
(**CHB;** Target:Neutral; 7.8e-3, Target: NonExon; 9.3e-3, Neutral:NonExon; .5713)

Pi P-values:
(**YRI;** Target:Neutral; 2.0e-3, Target: NonExon; 5.2e-3, Neutral:NonExon; 2.0e-2)
(**CEU;** Target:Neutral; 7.8e-3, Target: NonExon; 1.2e-2, Neutral:NonExon; 0.1958)
(**CHB;** Target:Neutral; 1.4e-3, Target: NonExon; 3.1e-3, Neutral:NonExon; 7.1e-2)

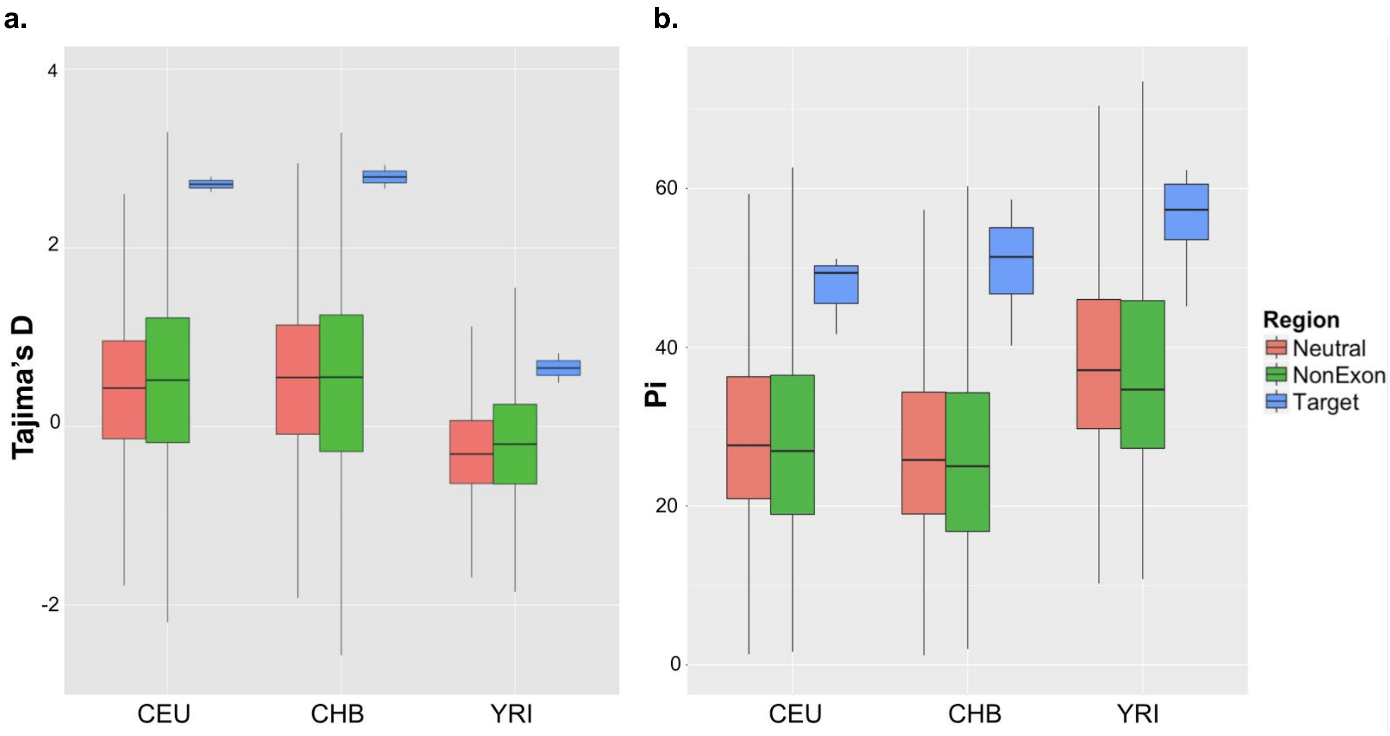

Figure S8. The regulatory potential in the *LCE3BC haplotype block.* The UCSC genome browser screenshot depicts the chromosomal location of the *LCE3BC* deletion (red bracket). The grey circle shows the deletion overlapping with multiple transcription factor binding sites. The blue arrow show peaks of conservation among vertebrates that coincides with LCE3C and LCE3B.

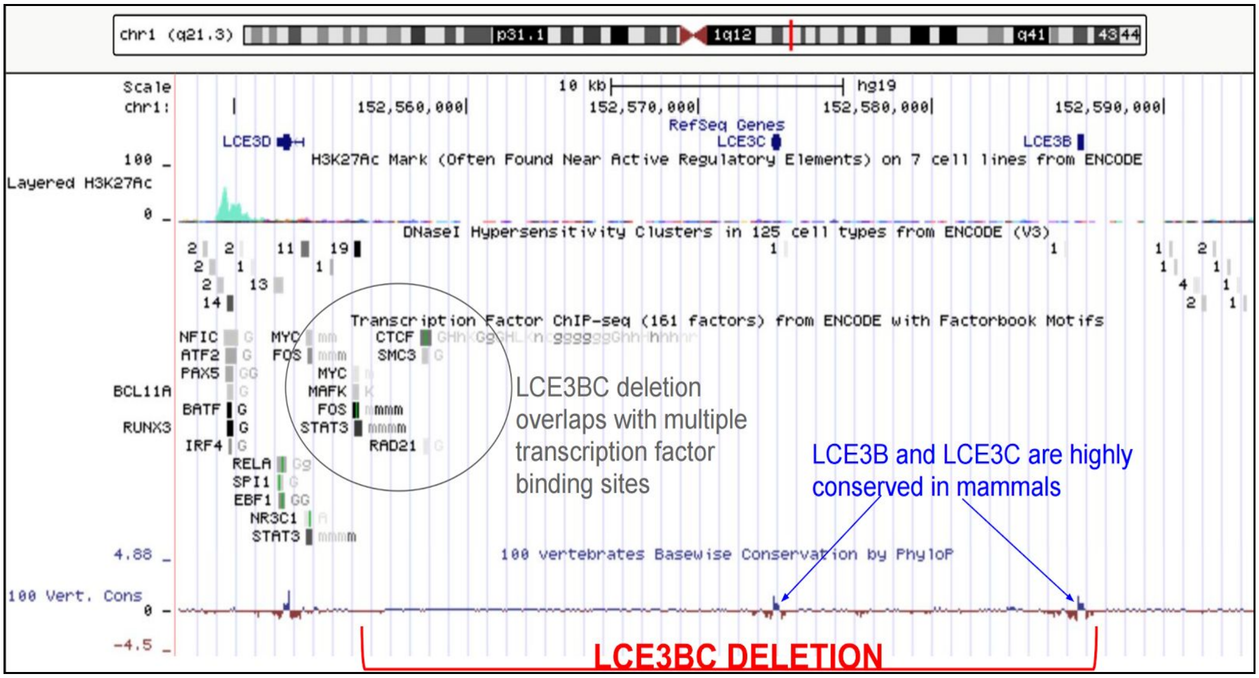

Figure S9. Gene expression levels (y-axis) of *LCE3A*, in sun-exposed skin in the lower leg, for individuals Homozygous for no deletion, Heterozygous, and Homozygous with the deletion. Data was displayed from GTex Portal. The SNP rs6693105 (high LD w/ deletion) was used to for this analysis. The effect size= 0.4, p=$6.4x10^{-10}$

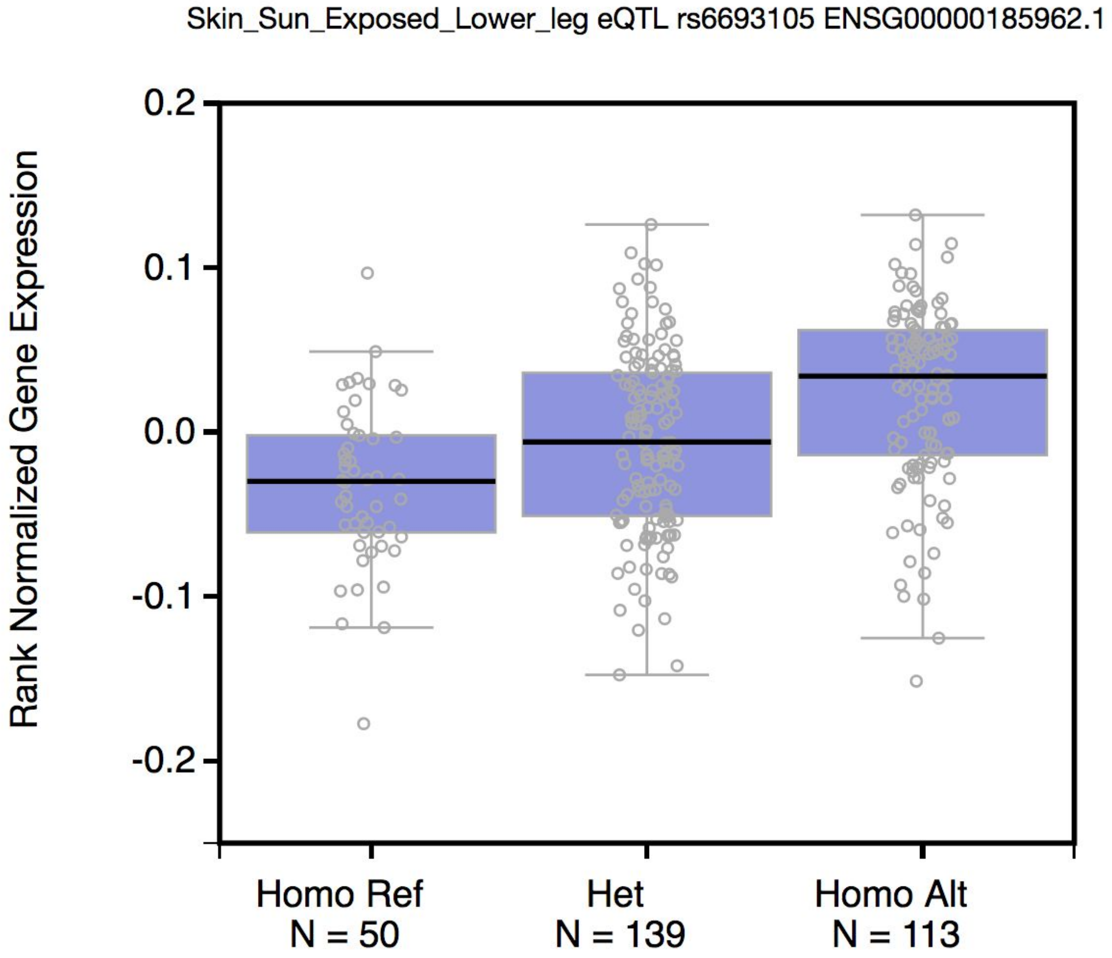

Supplement: Additional file 1: — Supplementary Figures. (PDF 1236 kb) [file 12862_2016_842_MOESM1_ESM.pdf]
